# Compounds from the Fruits of the Popular European Medicinal Plant *Vitex agnus-castus* in Chemoprevention via NADP(H):Quinone Oxidoreductase Type 1 Induction

**DOI:** 10.1155/2013/432829

**Published:** 2013-04-03

**Authors:** Shenghong Li, Shengxiang Qiu, Ping Yao, Handong Sun, Harry H. S. Fong, Hongjie Zhang

**Affiliations:** ^1^State Key Laboratory of Phytochemistry and Plant Resources in West China, Kunming Institute of Botany, The Chinese Academy of Sciences, Kunming, Yunnan 650204, China; ^2^South China Botanical Garden, Chinese Academy of Sciences, 723 Xingke Road, Tianhe District, Guangzhou 510650, China; ^3^Division of Life Science, The Hong Kong University of Science and Technology, Clear Water Bay Road, Hong Kong; ^4^Department of Medicinal Chemistry and Pharmacognosy, College of Pharmacy, University of Illinois at Chicago, 833 S. Wood Street, Chicago, IL 60612, USA; ^5^School of Chinese Medicine, Hong Kong Baptist University, 7 Baptist University Road, Kowloon Tong, Hong Kong

## Abstract

As part of our continuing efforts in the search for potential biologically active compounds from medicinal plants, we have isolated 18 compounds including two novel nitrogen containing diterpenes from extracts of the fruits of *Vitex agnus-castus*. These isolates, along with our previously obtained novel compound vitexlactam A (**1**), were evaluated for potential biological effects, including cancer chemoprevention. Chemically, the nitrogenous isolates were found to be two labdane diterpene alkaloids, each containing an **α**, **β**-unsaturated **γ**-lactam moiety. Structurally, they were elucidated to be 9**α**-hydroxy-13(14)-labden-16,15-amide (**2**) and 6**β**-acetoxy-9**α**-hydroxy-13(14)-labden-15,16-amide (**3**), which were named vitexlactams B and C, respectively. The 15 known isolates were identified as vitexilactone (**4**), rotundifuran (**5**), 8-epi-manoyl oxide (**6**), vitetrifolin D (**7**), spathulenol (**8**), *cis*-dihydro-dehydro-diconiferylalcohol-9-O-**β**-D-glucoside (**9**), luteolin-7-O-glucoside (**10**), 5-hydroxy-3,6,7,4′-tetramethoxyflavone (**11**), casticin (**12**), artemetin (**13**), aucubin (**14**), agnuside (**15**), **β**-sitosterol (**16**), *p*-hydroxybenzoic acid (**17**), and *p*-hydroxybenzoic acid glucose ester (**18**). All compound structures were determined/identified on the basis of 1D and/or 2D NMR and mass spectrometry techniques. Compounds **6**, **8**, **9**, and **18** were reported from a *Vitex* spieces for the first time. The cancer chemopreventive potentials of these isolates were evaluated for NADP(H):quinone oxidoreductase type 1 (QR1) induction activity. Compound **7** demonstrated promising QR1 induction effect, while the new compound vitexlactam (**3**) was only slightly active.

## 1. Introduction

Botanicals are widely used as either dietary supplements or herbal medicines throughout the world for the prevention and mitigation against various diseases or ailments. Among these botanicals are plants of the genus *Vitex* plants. Botanically, this genus was previously placed in the family of Verbenaceae but was recently revised as belonging to the family Lamiaceae, which itself was formerly known as the Labiatae. *Vitex *consists of about 250 species distributed worldwide, but mainly in the tropical and temperate zones [1]. A number of species (e.g., *V. agnus-castus*, *V. trifolia*, *V. negundo*, and *V. rotundifolia*) have been used as traditional medicinal plants. To date, more than 20 *Vitex* species have been investigated for chemical and biological properties, with approximately 200 compounds, mainly flavonoids, terpenoids, steroids, iridoids, and lignans, having been isolated and characterized [2].

*Vitex agnus-castus* Linn., is commonly known as the chaste tree, grows to a height of 2-3 m, and is distributed in the Mediterranean Region, Central Asia, and Southern Europe [3]. It is also cultivated in the various regions including the United States [4]. The fruits of *V. agnus-castus* are popularly used as a phytomedicine in Europe for the treatment of female hormonal disorders [5–7]. The fruit extract is also used as an alternative phytotherapeutic agent in the treatment of mastalgia [8]. There has been extensive research conducted on this phytomedicine leading to a large library of published literature on the pharmacognosy, traditional uses, chemical constituents, biology/pharmacology, and clinical studies [9]. In a previous communication we reported the isolation, structure determination, and X-ray crystallographic analysis of a novel labdane diterpene lactam from the *n*-hexane extracts of the fruits of this plant [10]. Further phytochemical studies of both of the *n*-hexane and methanol extracts resulted in the isolation of two additional new labdane diterpene lactams (**2-3**) and fifteen known compounds (**4–18**). In this paper, we describe the isolation and structure characterization of the two new metabolites and the identification of the 15 known compounds, as well as evaluating their NADP(H):quinone oxidoreductase type 1 (QR1) induction activity potentials.

## 2. Materials and Methods

### 2.1. General Experimental Procedures

All melting points were measured on an XRC-1 micromelting point apparatus and are uncorrected. 1D (one-dimensional) and 2D (two-dimensional) NMR (nuclear magnetic resonance) experiments were performed either on a Bruker AM-400 or a Bruker DRX-500 spectrometer. Unless otherwise is specified, chemical shifts (*δ*) were expressed in ppm with reference to the solvent signals. FABMS (fast atom bombardment mass spectrometry) and HRFABMS (high resolution fast atom bombardment mass spectrometry) were taken on a VG Auto Spec-3000 or a Finnigan MAT 90 instrument. IR (infrared) spectra were recorded on a Bio-Rad FTS-135 spectrometer with KBr pellets. UV (ultraviolet) spectral data were obtained on a UV 210A spectrometer. Optical rotations were carried out on a HORIBA SEPA-300 High Sensitive Polarimeter or a Perkin-Elmer model 241 Polarimeter. Column chromatography was performed either on Si gel (silica gel) (200–300 mesh, Qingdao Marine Chemical Inc., China), Si gel H (10–40 *μ*, Qingdao Marine Chemical Inc., China), Diaion HP-20 (Shandong Lukang Pharmaceutical Co., Ltd., China), Chromatorex ODS (Fuji Silysia Chemical Corporation, Ltd., Japan), or Lichroprep Rp_18_ gel (40–63 *μ*m, Merck, Darmstadt, Germany). Fractions were monitored by silica gel TLC (thin layer chromatography) [CHCl_3_-Me_2_CO (chloroform-acetone) 9 : 1, 8 : 2, 7 : 3], and spots were visualized by heating silica gel plates sprayed with 10% H_2_SO_4_ in EtOH (ethanol).

### 2.2. Plant Material

The fruits of *V. agnus-castus* were purchased from Frontier Botanicals, Norway, IA, USA (Lot. No. 799. 0116).

### 2.3. Extraction and Isolation

Dried fruits of *V. agnus-castus* (4077 g) were milled and sequentially extracted with *n*-hexane (3 × 8 L) for 28 h and MeOH (methanol) (4 × 9 L) for 24 h. The *n*-hexane extract was filtered and concentrated *in vacuo* to dryness to afford 200 g of a residue (part I). The MeOH extract was filtered, concentrated, and diluted with water (2 L), followed by partitioning with EtOAc (ethyl acetate) (4 × 3 L). The organic layer was evaporated *in vacuo* to dryness to give 60 g of a residue (part II). The water-soluble fraction was chromatographed on a column of Diaion HP-20 eluting with aqueous MeOH (30%→80%→100%). The 80% MeOH-H_2_O fraction was concentrated *in vacuo* to yield 48 g of a dry residue (part III).

#### 2.3.1. Isolation

Part I (200 g) was absorbed on 200 g of silica gel and chromatographed on a prepacked (500 g) silica gel column, eluting stepwise with *n*-hexane, CHCl_3_, CHCl_3_-Me_2_CO/1 : 1, and Me_2_CO. Compound **16** (27 mg) was crystallized from the CHCl_3_ fraction and compound **11** (336 mg) was crystallized from the CHCl_3_-Me_2_CO/1 : 1 fraction. The remaining CHCl_3_-Me_2_CO/1 : 1 eluate was filtered (40 g, net weight) and subjected to further chromatographic separation over a Chromatorex ODS column (eluent: 80% MeOH-H_2_O as eluents) and silica gel columns (using *n*-hexane-CHCl_3_/1 : 2,* n*-hexane-EtOAc/3 : 2, and* n*-hexane-Me_2_CO/2 : 1 as eluents) to provide compounds **1** (40 mg), **2** (4 mg), **3** (11 mg), **4** (25 mg), **5** (67 mg), **6** (6 mg), **7** (14 mg), **8** (14 mg), and **13** (9 mg).

Part II (60 g) was absorbed on 100 g of silica gel and chromatographed on a prepacked (300 g) silica gel column, eluting with CHCl_3_-Me_2_CO (1 : 0, 9 : 1, 8 : 2, 7 : 3, 0 : 1). Compound **12** (1.635 g) was crystallized from the CHCl_3_-Me_2_CO/1 : 0-9 : 1 fraction. Part of the CHCl_3_-Me_2_CO/8 : 2 fraction (0.810 g) was further chromatographed on RP_18_ gel (100 g) with 40% aqueous MeOH as eluents to give compound **17** (125 mg).

Part III (48 g) was again chromatographed on a Chromatorex ODS column eluting with aqueous MeOH (30%) and over a silica gel column eluting with CHCl_3_-MeOH (3 : 1), CHCl_3_-MeOH-H_2_O (4 : 1 : 0.1), and EtOAc-MeOH (12 : 1) to yield compounds **9** (108 mg), **10** (23 mg), **14** (55 mg), **15** (60 mg), and **18** (15 mg).

### 2.4. Structural Characterization of Novel Isolates

#### 2.4.1. Vitexlactam B **(2)**

White crystals, m.p. 162°C, C_20_H_33_NO_2_; [*α*]_D_^23.5^ + 18.75° (*c* 0.2, CHCl_3_); IR (KBr) *ν*_max⁡_: 3473, 3187, 3055, 2924, 2682, 1684, 1648, 1442, 1379, 1296, 1254, 1228, 1140, 1085, 1057, 1041, 1018, 972, 962, 943, 909, 832, 791, 777, 698 cm^−1^; ^1^H NMR (500 MHz, CDCl_3_) *δ* 1.50 (1H, dd, *J* = 11.0, 2.0 Hz, H-5), 1.75 (1H, m, H-8), 1.78 (1H, m, H-11a), 1.67 (1H, m, H-11b), 2.36 (2H, *br* t, *J* = 8.2 Hz, H_2_-12), 6.69 (1H, *br* s, H-14), 3.89 (2H, *br* s, H_2_-15), 0.88 (3H, d, *J* = 6.6 Hz, H_3_-17), 0.85 (3H, s, H_3_-18), 0.80 (3H, s, H_3_-19), 0.90 (3H, s, H_3_-20), 6.61 (1H, *br *s, NH); ^13^C NMR data, see Table 1; EIMS (electron impact mass spectrum) *m/z* 319 [M]^+^ (81), 304 (7), 286 (8), 206 (7), 194 (19), 180 (100), 167 (75), 152 (11), 138 (47), 123 (17), 110 (81), 96 (86), 82 (58), 69 (72), 55 (97); HREIMS *m/z* found 319.2509 [M]^+^, calcd. (calculated) 319.2511.

#### 2.4.2. Vitexlactam C **(3)**

White crystals, m.p. 178°C, C_22_H_35_NO_4_; [*α*]_D_^18.7^  −  12.73° (*c* 0.55, CHCl_3_); IR (KBr) *ν*_max⁡_: 3364, 3297, 2925, 2867, 1711, 1670, 1465, 1426, 1383, 1362, 1271, 1256, 1228, 1203, 1152, 1125, 1097, 1039, 1024, 977, 953, 916, 849, 819 cm^−1^; ^1^H NMR (400 MHz, CDCl_3_) *δ* 1.31 (1H, *br* d, *J* = 13.2 Hz, H-3a), 1.13 (1H, dt, *J* = 2.7, 13.2 Hz, H-3b), 1.58 (1H, d, *J* = 2.0 Hz, H-5), 5.35 (1H, *br* d, *J* = 2.2 Hz, H-6), 2.10 (1H, m, H-8), 1.90 (1H, m, H-11a), 1.72 (1H, m, H-11b), 2.43 (2H, m, H_2_-12), 5.82 (1H, *br* s, H-14), 3.91 (2H, *br* s, H_2_-16), 0.87 (3H, d, *J* = 6.7 Hz, H_3_-17), 0.93 (3H, s, H_3_-18), 0.97 (3H, s, H_3_-19), 1.22 (3H, s, H_3_-20), 6.92 (1H, *br* s, NH), 2.03 (3H, s, 6-OAc); ^13^C NMR data, see Table 1; EIMS *m/z* 377 [M]^+^ (3), 317 (76), 302 (15), 284 (6), 260 (29), 242 (8), 222 (21), 202 (23), 187 (48), 167 (60), 150 (28), 133 (41), 119 (64), 110 (68), 96 (97), 83 (72), 69 (77), 55 (100); HREIMS *m/z* found 377.2547 [M]^+^, calcd. 377.2566.

### 2.5. Chemoprevention Evaluation: NAD(P)H:Quinone Oxidoreductase Type 1 (QR1) Assay

Test compounds were evaluated for their potential to induce quinone reductase type 1 (QR1) activity with Hepa 1c1c7 cells. The cells were plated in 96-well plates at a density of 2 × 10^4^ cells/mL in 190 *μ*L of *α*-MEM (minimum essential medium) containing 100 units/mL penicillin G sodium, 100 *μ*g/mL streptomycin sulfate, and 250 ng/mL amphotericin B supplemented with 10% fetal bovine serum at 37°C in a 5% CO_2_ atmosphere. After preincubation for 24 h, the medium was changed, and test compounds were added to afford a final concentration range of 2 to 20 **μ**g/mL, and then the cells were incubated for an additional 48 h. The medium was decanted, and the cells were incubated with 50 *μ*L of 0.8% digitonin and 2 mM EDTA (ethylenediaminetetraacetic acid) solution (pH 7.8) at 37°C for 10 min. Quinone reductase activity was determined by measuring the NAD(P)H-dependent menadiol mediated reduction of MTT [3-(4, 5-dimethylthiazol-2-yl)-2,5-diphenyltetrazolium bromide] to a blue formazan. Cytotoxicity was determined by crystal violet staining assay. Induction of QR activity was calculated by comparing the QR specific activity of agent-treated cells with that of vehicle solvent-treated cells. 4′-Bromoflavone with a CD value of 12.9 nM was used as a positive control. CD represents the concentration of a test compound required to double QR induction in comparison with the vehicle control.

### 2.6. Supporting Information Available

NMR and MS data of the known compounds are available as Supplementary Material online at http://dx.doi.org/10.1155/2013/432829.

## 3. Results and Discussion

### 3.1. Plant Extracts and Isolation of Compounds

**Scheme 1:**
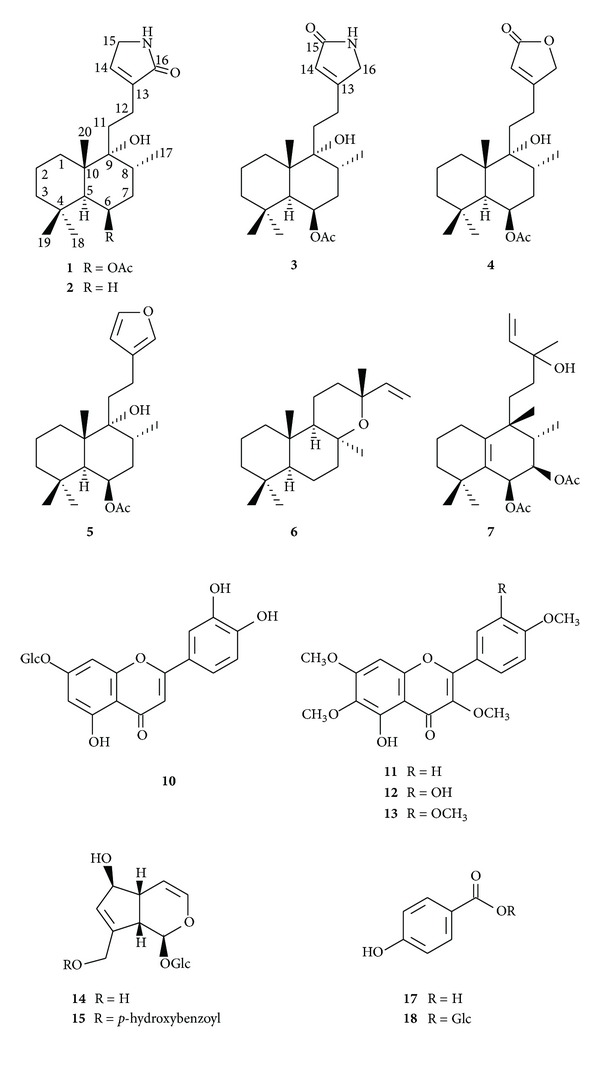


The purchased fruits of *V. agnus-castus* were milled and sequentially extracted with *n*-hexane and methanol. The *n*-hexane extract was successively chromatographed on silica gel and Chromatorex ODS to afford compounds **1–8**, **11**, **13**, and **16**. The methanol extract was partitioned between EtOAc and water. The EtOAc layer was chromatographed on silica gel to give compounds **12** and **17**. The water-soluble fraction was chromatographed on columns of Diaion HP-20, Chromatorex ODS, and silica gel to yield compounds **9**, **10**, **14**, **15**, and **18**  (Scheme 1).

### 3.2. Structure Elucidation and Identification of IsolatedCompounds

#### 3.2.1. Vitexlactam B **(2)**

Vitexlactam B (**2**) was obtained as white crystals. EI mass spectrum showed strong molecular ion peak at *m/z* 319 [M]^+^ (81% relative intensity), corresponding to a molecular formula of C_20_H_33_NO_2_, which was confirmed by high resolution EI mass spectrum (found: *m/z* 319.2509, calcd. 319.2511). The existence of a nitrogen atom was supported by its odd numbered molecular weight and a positive reaction to the Dragendorff reagent.

The ^1^H and ^13^C NMR (Table 1) spectra of **2**, being very similar to those of **1** [10], suggested that **2** is a closely related labdane diterpene alkaloid (Table 1), with an *α*, *β*-unsaturated *γ*-lactam moiety at the C-9 side chain. **2** differed from **1** only by the absence of the signals for an acetyl group and the replacement of an oxygen-bearing methine at *δ*_C_ 70.6 by a methylene signal at *δ*_C_ 21.7, indicating that **2** is the 6-deacetoxy derivative of **1**. The result was further supported by the facts that **1** was 58 atomic mass units less than **2** and the lack of an acetoxy group being observed in the IR spectrum of **2**. Full assignments of **2** using 2D NMR (including ^1^H-^1^H COSY (correlation spectroscopy), HMQC (heteronuclear multiple-quantum correlation spectroscopy), HMBC (heteronuclear multiple bond correlation spectroscopy), and ROESY (rotating-frame Overhauser spectroscopy)) techniques established the structure of **2** to be the expected 9*α*-hydroxy-13(14)-labden-16,15-amide. Compound **2** was accordingly identified as the deacetoxy derivative of **1** and was given the trivial name of vitexlactam B.

#### 3.2.2. Vitexlactam C **(3)**

**Figure 1:**
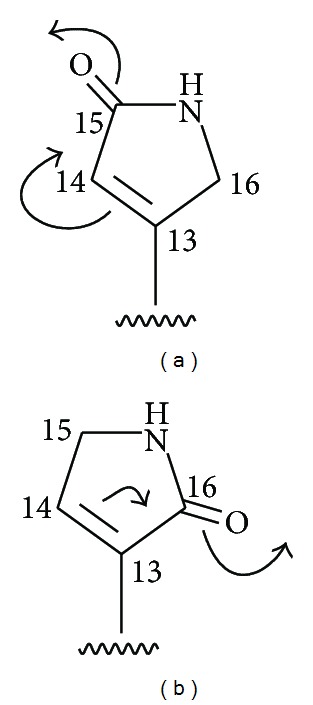
Electronic clouds movements of two different conjugated systems in compounds **3** (a) and **1** (b).

**Figure 2:**
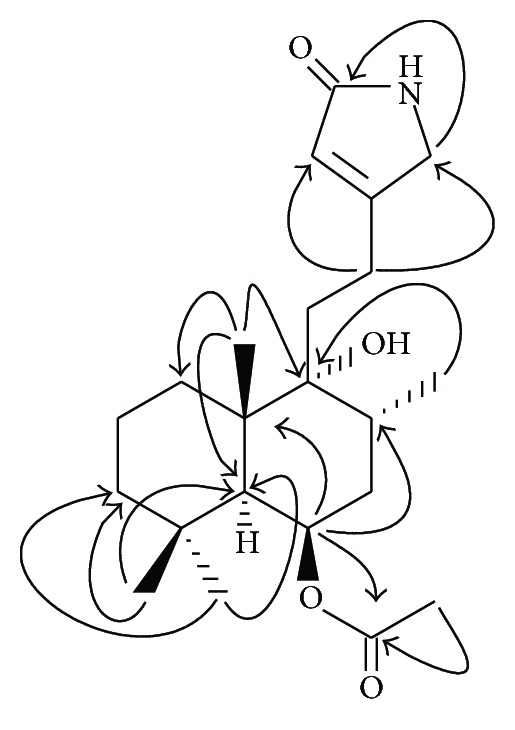
Key HMBC correlations of vitexlactam C (**3**).

**Figure 3:**
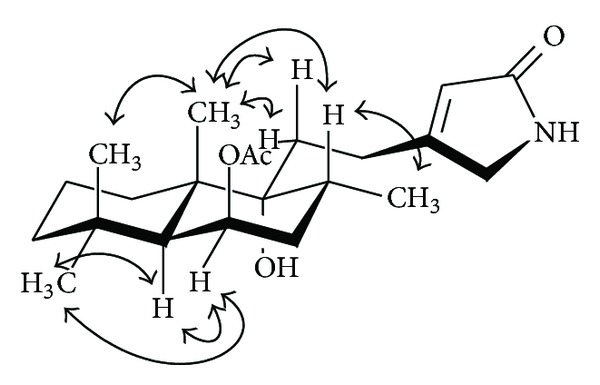
Key NOESY correlations of vitexlactam C (**3**).

Vitexlactam C (**3**) was also isolated as white crystals. EI mass spectrum under 70 eV displayed a weak [M]^+^ ion peak at *m/z* 377 (3%) identical with that of **1** in both the mass charge ratio and the relative intensity [11]. In addition, a strong fragment ion peak at *m/z* 317 (76%) due to [M-AcOH]^+^ and a series of fragment ions similar to those for **1** were also observed. High resolution EI mass spectrum (found: *m/z* 377.2547, calcd. 377.2566) established that both compounds have the same molecular formula of C_22_H_35_NO_4_. Therefore **3** was tentatively identified as an isomer of **1**. Comparison of the ^1^H and ^13^C NMR (Table 1) spectra of **3** with those of **1** (Table 1) indicated that the two compounds were equivalent not only in their skeletons but also in their oxygenation patterns. NMR spectral differences between these two compounds are mainly due to the *α*, *β*-unsaturated *γ*-lactam moieties in their C-9 side chains. The conjugate functionality occurred in **3** was deduced to be type (a) in contrast to type (b) in **1** (Figure 1). In the former conjugating system, C-13 is in a deshielded position while C-14 and H-14 are in a shielded position. On the contrary, in the latter (type (b)), C-13 is in a shielded position while C-14 and H-14 are in a deshielded position. Accordingly, C-13 of **3** moved downfield from *δ*_C_ 140.6 (s) in **1** to *δ*_C_ 163.6 (s), and C-14/H-14 of **3** shifted upfield from *δ*_C/H_ 137.1 (d)/6.71 (1H, *br* s) in **1** to *δ*_C/H_ 121.2 (d)/5.82 (1H, *br* s). 2D NMR analysis of **3** revealed that, unlike in **1**, the ^1^H-^1^H COSY correlation between H-14 and the nitrogen-bearing methylene at *δ*_H_ 3.91 (2H, *br* s) and the ^1^H-^13^C interaction (Figure 2) between H_2_-12 [*δ*_H_ 2.44 (2H, m)] and the lactam carbonyl carbon at *δ*_C_ 175.3 (s) disappeared while ^1^H-^13^C interaction between H_2_-12 and the nitrogen-occurring methylene at *δ*_C_ 50.5 (t) were observed, thus confirming the presence of a type (a) conjugate functionality in **3**. Other structural correlations, including key NOEs (nuclear Overhauser effects) (Figure 3) in **3**, were identical with those in **1**.

A detailed spectral comparison between **3** and vitexilactone (**4**) [11] was also carried out. The molecular weight of **3** is lower by 1 mass unit than that of **4**. Besides, **3** differed from **4** (Table 1) mainly by the upfield shifted H_2_-16 and C-16 signals (from *δ*_H/C_ 4.77 (2H, *br* d, *J* = 1.3 Hz)/73.4 (t) in **4** to *δ*_H/C_ 3.94 (2H, *br* s)/50.5 (t) in **3**) and the existence of an extra NH proton at *δ*_H_ 6.92 (1H, *br* s), indicating that an *α*,*β*-unsaturated *γ*-lactam moiety in **3** took the place of the *α*,*β*-unsaturated *γ*-lactone in **4**. Based on all the abovedescribed spectral features, compound **3** was consequently deduced to be 6*β*-acetoxy-9*α*-hydroxy-13(14)-labden-15,16-amide and was named vitexlactam C. 

Considering that only mild conditions were employed and that no nitrogen containing solvents and chromatographic materials were involved in the entire extraction and separation procedures, we postulate that compounds **1–3** are biogenetic amination products of their corresponding lactones (e.g., **3** was derived from **4**).

#### 3.2.3. Identification of Known Compounds

Along with the new compounds, fifteen known compounds were also isolated in the course of the current study. Through comparison of their ^1^H and ^13^C NMR and MS data with those values reported in the literature, they were identified as three labdane-type diterpenoids, vitexilactone (**4**) [11]; rotundifuran (**5**) [11], and 8-epi-manoyl oxide (**6**) [12] ([*α*]_D_^19.5^  −  11.8°; *c* = 0.55, CHCl_3_); a rearranged labdane (halimane) diterpenoid, vitetrifolin D (**7**) [13]; an aromadendrene-type sesquiterpenoid, spathulenol (**8**) [14, 15]; a lignan glucoside, *cis*-dihydro-dehydro-diconiferylalcohol-9-O-*β*-D-glucoside (**9**) [16]; four flavonoids, luteolin-7-O-glucoside (**10**) [17], 5-hydroxy-3,6,7,4′-tetramethoxyflavone (**11**) [18], casticin (**12**) [19], and artemetin (**13**) [20]; two iridoid glycosides, aucubin (**14**) [21] and agnuside (**15**) [22]; a sterol, *β*-sitosterol (**16**) (comparison with an authentic sample); and two simple phenolics, *p*-hydroxybenzoic acid (**17**) [22] and *p*-hydroxybenzoic acid glucose ester (**18**) [22]. The occurrence of compounds **7–9** and **18** in the genus *Vitex* is being reported for the first time.

### 3.3. Activity Evaluation of the Isolated Compounds on QR1 Induction

These compounds have been evaluated for their potential chemopreventive activity by induction of the ubiquitous flavoenzyme NADP(H):quinone oxidoreductase type 1 (QR1) with cultured Hepa 1c1c7 cells. QR1 has been determined as an important phase II detoxification enzyme that can protect cells against the harmful effects caused by free radicals and reactive oxygen species by catalyzing the reduction of quinones to hydroquinones [23]. Hence, enhanced activity of the enzyme provides protection of cells from potential carcinogenicity. Vitetrifolin D (**7**) was shown to induce QR1 activity with a CD value of 23.2 *μ*M. Although vitexlactam C (**3**) induced QR1 by 1.5 times that of the vehicle control at a concentration of 5.3 *μ*M, it was toxic to Hepa 1c1c7 cells with 57% inhibition of the cells at 26.5 *μ*M. None of the other compounds demonstrated QR1 induction activity.

## 4. Conclusion

The fruits of *Vitex agnus-castus* have been popularly used as a phytomedicine in Europe, especially Germany, for the treatment of premenstrual stress syndrome. However, the evaluation of this herb or its phytochemical constituents for cancer chemoprevention activity has not been reported. Thus, we undertook a study of the 18 compounds we isolated from the fruits of this plant in a bioassay, which have been used for assessing chemoprevention potentials. The isolates, including several novel nitrogen containing labdane diterpenes, were thus evaluated for their potentials in the induction of the phase II detoxification enzyme QR1. Results showed that only the labdane compounds **3** and **7** demonstrated QR1 induction effect. We have demonstrated that compounds possessing potential chemopreventive action do exist in *V. agnus-castus* and that further phytochemical and biological investigations of this plant material coupled with structure modification studies are needed in order to discover additional/modified labdanes possessing more potent QR1 induction activity and chemopreventive potential.

## Supplementary Material

NMR and MS data of the known compounds from *Vitex agnus-castus*.

## Figures and Tables

**Table 1:** ^
13^C NMR data of compounds **1**–**7** (CDCl_3_, *δ* in ppm).

Carbon	**1** ^ a^	**2** ^ b^	**3** ^ a^	**4** ^ a^	**5** ^ a^	**6** ^ a^	**7** ^ b^
C-1	33.7 t	32.5 t	33.7 t	33.8 t	33.9 t	36.5 t	25.9 t
C-2	18.8 t	18.7 t	18.6 t	18.9 t	18.7 t	20.7 t	19.4 t
C-3	43.8 t	41.7 t	43.6 t	43.9 t	43.7 t	42.3 t	39.4 t
C-4	33.9 s	33.3 s	34.0 s	34.3 s	34.8 s	32.9 s	34.6 s
C-5	47.5 d	46.2 d	47.6 d	48.0 d	47.5 d	46.3 d	132.5 s
C-6	70.6 d	21.7 t	69.9 d	70.1 d	70.3 d	21.0 t	66.2 d
C-7	36.3 t	31.4 t	36.1 t	36.4 t	36.1 t	37.9 t	72.7 d
C-8	32.1 d	36.8 d	31.9 d	32.3 d	33.6 d	74.1 s	36.4 d
C-9	76.4 s	76.8 s	76.7 s	76.8 s	76.8 s	61.2 d	42.9 s
C-10	44.0 s	43.3 s	43.8 s	44.1 s	43.7 s	38.9 s	141.5 s
C-11	32.3 t	32.0 t	32.3 t	31.9 t	31.8 t	18.6 t	29.3 t
C-12	21.7 t	22.0 t	26.5 t	25.7 t	21.5 t	45.1 t	38.6 t
C-13	140.6 s	140.8 s	163.6 s	171.3 s	125.5 s	73.6 s	73.0 s
C-14	137.1 d	136.9 d	121.2 d	115.3 d	110.8 d	146.1 d	144.5 d
C-15	46.6 t	46.4 t	175.3 s	171.3 s	142.9 d	111.1 t	112.1 t
C-16	175.3 s	175.8 s	50.5 t	73.4 t	138.5 d	27.4 q	27.8 q
C-17	16.4 q	16.6 q	16.0 q	16.3 q	16.1 q	32.0 q	11.1 q
C-18	33.6 q	33.7 q	33.6 q	33.8 q	33.6 q	33.1 q	29.3 q
C-19	23.7 q	22.1 q	23.6 q	23.9 q	23.7 q	21.3 q	28.1 q
C-20	18.9 q	16.2 q	19.0 q	19.2 q	19.0 q	24.7 q	28.0 q
OAc	170.5 s		170.3 s	170.6 s	170.7 s		170.8
(C=O)							(2C, s)
OAc	21.9 q		21.8 q	22.1 q	21.9 q		21.4 q
(CH_3_)							20.9 q

^a^Recorded at 100 MHz.

^b^Recorded at 125 MHz.
