# Supplementary material for: Compounds from the Fruits of the Popular European Medicinal Plant Vitex agnus-castus in Chemoprevention via NADP(H):Quinone Oxidoreductase Type 1 Induction
Source: Evid Based Complement Alternat Med. 2013 Apr 3;2013:432829. doi: 10.1155/2013/432829 (PMC3638617; doi:10.1155/2013/432829)
Supplement: Supplementary file 1 — NMR and MS data of the known compounds from Vitex agnus-castus. [file 432829.f1.pdf]

Supporting Information

NMR and MS data of the known compounds from *Vitex agnus-castus*

Vitexlactam A (**1**): white crystals, mp. 207°C, $C_{22}H_{35}NO_4$; $[\alpha]_D^{23.7}$ -10.71° (*c* 0.42, $CHCl_3$); IR (KBr) ν_{max}: 3463, 3188, 3054, 2942, 2923, 1731, 1684, 1460, 1442, 1381, 1361, 1250, 1226, 1142, 1087, 1039, 1020, 973, 962, 952, 913, 832, 781, 749, 645, 610, 598 cm^{-1}; ^1H NMR (400 MHz, $CDCl_3$) δ see Table 3-3; ^{13}C NMR (100 MHz, $CDCl_3$) δ see Table 3-4; EIMS *m/z* 377 $[M]^+$ (3), 317 (87), 302 (5), 284 (17), 206 (13), 192 (15), 180 (72), 167 (100), 149 (18), 138 (74), 123 (22), 110 (70), 96 (56), 82 (40), 69 (50), 55 (67); HREIMS *m/z* found 377.2569 $[M]^+$, calcd. 377.2566.

Vitexlactam B (**2**): white crystals, mp. 162°C, $C_{20}H_{33}NO_2$; $[\alpha]_D^{23.5}$ +18.75° (*c* 0.2, $CHCl_3$); IR (KBr) ν_{max}: 3473, 3187, 3055, 2924, 2682, 1684, 1648, 1442, 1379, 1296, 1254, 1228, 1140, 1085, 1057, 1041, 1018, 972, 962, 943, 909, 832, 791, 777, 698 cm^{-1}; ^1H NMR (500 MHz, $CDCl_3$) δ see Table 3-3; ^{13}C NMR (125 MHz, $CDCl_3$) δ see Table 3-4; EIMS *m/z* 319 $[M]^+$ (81), 304 (7), 286 (8), 206 (7), 194 (19), 180 (100), 167 (75), 152 (11), 138 (47), 123 (17), 110 (81), 96 (86), 82 (58), 69 (72), 55 (97); HREIMS *m/z* found 319.2509 $[M]^+$, calcd. 319.2511.

Vitexlactam C (**3**): white crystals, mp. 178°C, $C_{22}H_{35}NO_4$; $[\alpha]_D^{18.7}$ -12.73° (c 0.55, $CHCl_3$); IR (KBr) ν_{max}: 3364, 3297, 2925, 2867, 1711, 1670, 1465, 1426, 1383, 1362, 1271, 1256, 1228, 1203, 1152, 1125, 1097, 1039, 1024, 977, 953, 916, 849, 819 cm^{-1}; ^1H NMR (400 MHz, $CDCl_3$) δ see Table 3-3; ^{13}C NMR (100 MHz, $CDCl_3$) δ see Table 3-4; EIMS *m/z* 377 $[M]^+$ (3), 317 (76), 302 (15), 284 (6), 260 (29), 242 (8), 222 (21), 202 (23), 187 (48), 167 (60), 150 (28), 133 (41), 119 (64), 110 (68), 96 (97), 83 (72), 69 (77), 55 (100); HREIMS *m/z* found 377.2547 $[M]^+$, calcd. 377.2566.

Vitexilactone (**4**): colorless lamellar crystals, $C_{22}H_{34}O_5$; ^1H NMR (400 MHz, $CDCl_3$) δ see Table 3-2; ^{13}C NMR (100 MHz, $CDCl_3$) δ see Table 3-4; EIMS *m/z* 318 [M-

$CH_3COOH]^+$ (100), 303 (18), 285 (3), 275 (7), 262 (9), 221 (5), 207 (11), 194 (21), 181 (40), 168 (32), 150 (35), 135 (34), 123 (45), 109 (50), 95 (42), 81 (45), 69 (65), 55 (57).

Rotundifuran (**5**): colorless oil, $C_{22}H_{34}O_4$; ^{1}H NMR (400 MHz, $CDCl_3$) δ see Table 3-2; ^{13}C NMR (100 MHz, $CDCl_3$) δ see Table 3-4; EIMS *m/z* 362 $[M]^+$ (7), 318 (18), 302 (81), 287 (17), 269 (8), 221 (12), 207 (21), 189 (20), 177 (33), 165 (54), 150 (89), 135 (72), 123 (100), 109 (77), 95 (69), 81 (80), 69 (61), 55 (53).

8-Epimanoyl oxide (**6**): colorless needles, $C_{20}H_{34}O$; $[\alpha]_D^{19.5}$ -11.82° (*c* 0.55, $CHCl_3$); ^{1}H NMR (400 MHz, $CDCl_3$) δ see Table 3-2; ^{13}C NMR (100 MHz, $CDCl_3$) δ see Table 3-4; EIMS *m/z* 290 $[M]^+$ (35), 272 (20), 257 (18), 247 (12), 232 (10), 219 (16), 203 (27), 192 (40), 177 (60), 163 (35), 149 (40), 137 (55), 123 (61), 109 (67), 95 (70), 81 (71), 69 (80), 55 (100).

Vitetrifolin D (**7**): colorless oil, $C_{24}H_{38}O_5$; $[\alpha]_D^{19.1}$ +139.58° (*c* 0.6, $CHCl_3$); IR (KBr) ν_{max}: 3499, 2967, 2928, 2865, 1740, 1465, 1369, 1249, 1028, 978, 941, 920, 874, 743, 695, 484 cm^{-1}; ^{1}H NMR (400 MHz, $CDCl_3$) δ see Table 3-1; ^{13}C NMR (100 MHz, $CDCl_3$) δ see Table 3-4; EIMS *m/z* 346 $[M\text{-}CH_3COOH]^+$ (31), 328 (23), 304 (28), 286 (20), 271 (26), 248 (24), 201 (27), 188 (33), 178 (30), 136 (33), 121 (100), 109 (30), 95 (33), 79 (32), 69 (36), 55 (41).

Spathulenol (**8**): $C_{15}H_{24}O$; ^{1}H NMR (400 MHz, $CDCl_3$) δ 2.22 (1H, m, H-1α), 0.46 (1H, dd, *J* = 9.5, 11.3 Hz, H-6α), 0.73 (1H, m, H-7α), 2.41 (1H, dd, *J* = 7.4, 16.5 Hz, H-9β), 1.03 (3H, s, H-12), 1.05 (3H, s, H-13), 4.68 (1H, s, H-14a), 4.66 (1H, s, H-14b), 1.27 (3H, s, H-15); ^{13}C NMR (125 MHz, $CDCl_3$) δ 53.4 (d, C-1), 26.7 (t, C-2), 41.7 (t, C-3), 81.0 (s, C-4), 54.3 (d, C-5), 29.9 (d, C-6), 27.4 (d, C-7), 24.8 (t, C-8), 38.8 (t, C-9), 153.4 (s, C-10), 20.2 (s, C-11), 16.3 (q, C-12), 28.6 (q, C-13), 106.2 (t, C-14), 26.0 (q, C-15); Positive FABMS *m/z* 203 (100) $[M\text{-}H_2O]^+$, 187 (4), 175 (6), 159 (15), 147 (14), 138 (8), 113 (2), 99 (9), 84 (2), 59 (3).

Cis-dihydro-dehydro-diconiferylalcohol-9-O-β-D-glucoside (**9**): colorless syrup, $C_{26}H_{34}O_{11}$; ^{1}H NMR (400 MHz, CD_3OD) δ 6.99 (1H, d, *J* = 1.7 Hz, H-2), 6.75 (1H, d, *J* = 8.0 Hz, H-5), 6.85 (1H, dd, *J* = 1.8, 8.1 Hz, H-6), 5.57 (1H, d, *J* = 6.5 Hz, H-7), 3.65 (1H, m, H-8), 4.10 (1H, t, *J* = 8.0 Hz, H-9a), 3.83 (1H, overlap, H-9b), 6.71 (1H, s, H-2′), 6.79 (1H, s, H-6′), 2.61 (2H, t, *J* = 7.3 Hz, H_2-7′), 1.80 (2H, m, H_2-8′), 3.56 (2H, t, *J* = 6.5

Hz, H_2-9′), 4.35 (1H, d, *J* = 7.8 Hz, H-1″), 3.24 (1H, t, *J* = 8.8 Hz, H-2″), 3.36 (1H, t, *J* = 8.8 Hz, H-3″), 3.30 (1H, overlap, H-4″), 3.28 (1H, m, H-5″), 3.87 (1H, dd, *J* = 2.0, 12.2 Hz, H-6a), 3.68 (1H, dd, *J* = 5.4, 11.9 Hz, H-6b), 3.81 (3H, s, 3-OCH_3), 3.85 (3H, s, 3′-OCH_3); ^{13}C NMR (100 MHz, CD_3OD) δ 134.6 (s, C-1), 111.0 (d, C-2), 149.0 (s, C-3), 147.4 (s, C-4), 116.1 (d, C-5), 119.8 (d, C-6), 99.1 (d, C-7), 52.8 (d, C-8), 72.3 (t, C-9), 136.9 (s, C-1′), 114.3 (d, C-2′), 145.1 (s, C-3′), 147.4 (s, C-4′), 129.7 (s, C-5′), 118.2 (d, C-6′), 32.8 (t, C-7′), 35.9 (t, C-8′), 62.3 (t, C-9′), 104.2 (d, C-1″), 75.1 (d, C-2″), 78.2 (d, C-3″), 71.6 (d, C-4″), 77.9 (d, C-5″), 62.7 (t, C-6″), 56.9 (q, 3′-OCH_3), 56.5 (q, 3-OCH_3); Negative FABMS *m/z* 522 $[M]^+$ (27), 357 (12), 341 (100), 327 (29), 311 (14), 297 (7), 179 (54), 119 (37).

Luteolin-7-O-glucoside (**10**): yellow needles, $C_{21}H_{20}O_{11}$; ^{1}H NMR (500 MHz, DMSO-d_6) δ 6.74 (1H, s, H-3), 6.43 (1H, d, *J* = 2.0 Hz, H-6), 6.78 (1H, d, *J* = 2.5 Hz, H-8), 7.40 (1H, d, *J* = 2.0 Hz, H-2′), 6.89 (1H, d, *J* = 8.0 Hz, H-5′), 7.44 (1H, dd, *J* = 2.5, 8.0 Hz, H-6′), 5.06 (1H, d, *J* = 7.5 Hz, H-1″), 3.17 (1H, t, *J* = 8.5, H-2″), 3.40 (1H, overlapped by water, H-3″), 3.29 (1H, overlap, H-4″), 3.25 (1H, m, H-5″), 3.70 (1H, d, *J* = 10.5 Hz, H-6″a), 3.43 (1H, overlapped by water, H-6″b), 12.96 (1H, s, 5-OH); ^{13}C NMR (125 MHz, DMSO-d_6) δ 164.6 (s, C-2), 103.2 (d, C-3), 182.0 (s, C-4), 161.2 (s, C-5), 99.6 (d, C-6), 163.0 (s, C-7), 94.8 (d, C-8), 157.0 (s, C-9), 105.4 (s, C-10), 121.4 (s, C-1′), 113.6 (d, C-2′), 145.8 (s, C-3′), 150.0 (s, C-4′), 116.1 (d, C-5′), 119.3 (d, C-6′), 100.0 (d, C-1″), 73.2 (d, C-2″), 77.2 (d, C-3″), 69.6 (d, C-4″), 76.4 (d, C-5″), 60.7 (t, C-6″); Negative FABMS *m/z* 447 $[M-H]^+$ (100), 433 (10), 325 (9), 285 (34), 202 (4).

5-Hydroxy-3, 6, 7, 4′-tetramethoxyflavone (**11**): pale yellow blocks, $C_{19}H_{18}O_7$; ^{1}H NMR (500 MHz, $CDCl_3$) δ 6.48 (1H, s, H-8), 8.05 (2H, dd, *J* = 3.0, 12.0 Hz, H-2′ and H-6′), 7.00 (2H, dd, *J* = 2.5, 11.5 Hz, H-3′ and H-5′), 12.6 (1H, s, 5-OH), 3.94, 3.91, 3.88 and 3.85 (each 3H, s, 4×OCH_3); ^{13}C NMR (100 MHz, $CDCl_3$) δ 152.2 (s, C-2), 138.6 (s, C-3), 178.8 (s, C-4), 152.7 (s, C-5), 132.3 (s, C-6), 158.7 (s, C-7), 90.3 (d, C-8), 155.9 (s, C-9), 106.5 (s, C-10), 122.7 (s, C-1′), 130.0 (2C, d, C-2′ and C-6′), 114.0 (2C, d, C-3′ and C-5′), 161.7 (s, C-4′), 60.7, 60.0, 56.2 and 55.3 (each q, 4×OCH_3); EIMS *m/z* 358 $[M]^+$ (100), 343 (56), 339 (26), 328 (8), 315 (13), 300 (8), 297 (8), 285 (6), 272 (3), 229 (3), 201 (4), 181 (10), 153 (12), 135 (25), 119 (13), 107 (8), 92 (9), 77 (17), 69 (20).

Casticin (**12**): $C_{19}H_{18}O_8$; ^1H NMR (400 MHz, $CDCl_3$) δ 6.44 (1H, s, H-8), 7.63 (1H, d, *J* = 2.2 Hz, H-2′), 6.89 (1H, d, *J* = 8.4 Hz, H-5′), 7.64 (1H, dd, *J* = 2.2, 10.9 Hz, H-6′), 3.92, 3.90, 3.87 and 3.81 (each 3H, s, 4×OCH_3); ^{13}C NMR (100 MHz, $CDCl_3$) δ 152.3 (s, C-2), 139.0 (s, C-3), 178.9 (s, C-4), 152.7 (s, C-5), 132.3 (s, C-6), 158.8 (s, C-7), 90.4 (d, C-8), 155.6 (s, C-9), 106.6 (s, C-10), 123.5 (s, C-1′), 110.5 (d, C-2′), 145.6 (s, C-3′), 148.9 (s, C-4′), 114.4 (d, C-5′), 121.5 (d, C-6′), 60.8, 60.1, 56.3 and 56.0 (each q, 4×OCH_3); EIMS *m/z* 374 $[M]^+$ (100), 359 (65), 345 (9), 331 (16), 316 (7), 301 (8), 285 (5), 273 (8), 245 (5), 217 (5), 181 (13), 164 (16), 151 (23), 135 (16), 121 (11), 109 (8), 93 (13), 77 (14), 69 (33).

Artemetin (**13**): yellow needles, $C_{20}H_{20}O_8$; ^1H NMR (400 MHz, $CDCl_3$) δ 6.49 (1H, s, H-8), 7.67 (1H, d, *J* = 2.0 Hz, H-2′), 6.98 (1H, d, *J* = 8.4 Hz, H-5′), 7.72 (1H, dd, *J* =2.0, 8.8 Hz, H-6′), 12.6 (1H, *br* s, 5-OH), 3.85, 3.91, 3.95, 3.96 and 3.97 (each 3H, s, 5×OCH_3); EIMS *m/z* 388 $[M]^+$ (100), 373 (58), 165 (15).

Aucubin (**14**): colorless syrup, $C_{15}H_{22}O_9$; ^1H NMR (400 MHz, CD_3OD) δ 4.94 (1H, d, *J* = 7.0 Hz, H-1), 6.30 (1H, dd, *J* = 2.0, 6.1 Hz, H-3), 5.08 (1H, dd, *J* = 2.0, 6.1 Hz, H-4), 2.65 (1H, m, H-5), 4.43 (1H, *br* dd, *J* = 1.6, 3.2 Hz, H-6), 5.76 (1H, *br* s, H-7), 2.89 (1H, *br* t, *J* = 7.4 Hz, H-9), 4.34 (1H, d, *J* = 15.3 Hz, H-10a), 4.16 (1H, d, *J* = 15.4 Hz, H-10b), 4.67 (1H, d, *J* = 8.0 Hz, H-1′), 3.22 (1H, t, *J* = 8.4 Hz, H-2′), 3.39 (1H, t, *J* = 8.6 Hz, H-3′), 3.30 (1H, overlap, H-4′), 3.28 (1H, m, H-5′), 3.85 (1H, dd, *J* = 1.2, 12.2 Hz, H-6′a), 3.64 (1H, dd, *J* = 5.4, 12.1 Hz, H-6′b); ^{13}C NMR (100 MHz, CD_3OD) δ 97.8 (d, C-1), 141.5 (d, C-3), 105.7 (d, C-4), 46.1 (d, C-5), 82.8 (d, C-6), 130.3 (d, C-7), 147.9 (s, C-8), 47.9 (d, C-9), 61.3 (t, C-10), 100.0 (d, C-1′), 74.8 (d, C-2′), 78.1 (d, C-3′), 71.5 (d, C-4′), 77.8 (d, C-5′), 62.6 (t, C-6′); Negative FABMS *m/z* 345 $[M-H]^+$ (5), 339 (90), 325 (100), 311 (61), 239 (4), 197 (4), 157 (3), 119 (2), 80 (15).

Agnuside (**15**): white powders, $C_{22}H_{26}O_{11}$; ^1H NMR (400 MHz, CD_3OD) δ 4.98 (1H, d, *J* = 7.4 Hz, H-1), 6.33 (1H, dd, *J* = 2.0, 6.0 Hz, H-3), 5.11 (1H, dd, *J* = 3.8, 6.1 Hz, H-4), 2.70 (1H, m, H-5), 4.46 (1H, *br* dd, *J* = 1.5, 3.6 Hz, H-6), 5.82 (1H, *br* s, H-7), 2.98 (1H, *br* t, *J* = 7.4 Hz, H-9), 5.08 (1H, d, *J* = 15.7 Hz, H-10a), 4.90 (1H, d, *J* = 15.7 Hz, H-10b), 4.70 (1H, d, *J* = 7.7 Hz, H-1′), 3.25 (1H, t, *J* = 9.0 Hz, H-2′), 3.39 (1H, t, *J* = 8.9 Hz, H-3′), 3.31 (1H, overlap, H-4′), 3.27 (1H, m, H-5′), 3.85 (1H, dd, *J* = 1.9, 12.2 Hz, H-6′a),

3.66 (1H, dd, *J* = 5.3, 12.0 Hz, H-6′b), 7.90 (2H, dd, *J* = 2.0, 8.8 Hz, H-3″ and H-7″), 6.83 (2H, dd, *J* = 2.0, 8.8 Hz, H-4″ and H-6″); ^{13}C NMR (100 MHz, CD_3OD) δ 98.0 (d, C-1), 141.7 (d, C-3), 105.5 (d, C-4), 46.2 (d, C-5), 82.8 (d, C-6), 132.5 (d, C-7), 142.8 (s, C-8), 48.8 (d, C-9), 63.6 (t, C-10), 100.3 (d, C-1′), 74.9 (d, C-2′), 77.9 (d, C-3′), 71.5 (d, C-4′), 78.1 (d, C-5′), 62.8 (t, C-6′), 167.8 (s, C-1″), 122.2 (s, C-2″), 132.9 (2C, d, C-3″ and C-7″), 116.3 (2C, d, C-4″ and C-6″), 163.6 (s, C-5″); Negative FABMS *m/z* 466 $[M-H]^+$ (100), 447 (4), 404 (10), 384 (4), 339 (51), 325 (44), 250 (3), 116 (2), 94 (2).

β-Sitosterol (**16**): colorless needles, $C_{29}H_{50}O$; EIMS *m/z* 414 $[M]^+$ (100), 396 (54), 381 (32), 369 (14), 351 (14), 329 (36), 314 (12), 303 (46), 289 (10), 273 (34), 255 (43), 231 (21), 213 (32), 173 (17), 159 (34), 145 (38), 119 (30), 107 (43), 95 (39), 81 (42), 69 (39), 55 (56).

***p*-Hydroxybenzoic acid** (**17**): white plates, $C_7H_6O_3$; ^{1}H NMR (400 MHz, Acetone-d_6) δ 7.92 (2H, d, *J* = 8.6 Hz, H-3 and H-7), 6.92 (2H, d, *J* = 8.6 Hz, H-4 and H-6); ^{13}C NMR (100 MHz, Acetone-d_6) δ 169.0 (s, C-1), 122.3 (s, C-2), 132.8 (2C, d, C-3 and C-7), 116.0 (2C, d, C-4 and C-6), 162.7 (s, C-5); EIMS *m/z* 138 $[M]^+$ (95), 121 (100), 110 (22), 93 (75), 81 (40), 74 (28), 65 (86), 53 (84).

***p*-Hydroxybenzoic acid glucose ester** (**18**): white powders, $C_{13}H_{16}O_8$; ^{1}H NMR (400 MHz, Pyridine-d_5) δ 8.24 (2H, d, *J* = 8.5 Hz, H-3 and H-7), 7.15 (2H, d, *J* = 8.5 Hz, H-4 and H-6), 6.65 (1H, d, *J* = 6.3 Hz, H-1′), 4.37-4.54 (6H, m, H-2′ to H-6′); ^{13}C NMR (100 MHz, Pyridine-d_5) δ 165.8 (s, C-1), 121.2 (s, C-2), 132.9 (2C, d, C-3 and C-7), 116.2 (2C, d, C-4 and C-6), 164.0 (s, C-5), 96.4 (d, C-1′), 74.5 (d, C-2′), 78.7 (d, C-3′), 71.2 (d, C-4′), 79.6 (d, C-5′), 62.3 (t, C-6′); Negative FABMS *m/z* 283 $[M-OH]^+$ (7), 245 (45), 212 (18), 186 (6), 116 (6), 79 (33).
